# Personalized nutrition and precision medicine in perimenopausal women: A minireview of genetic polymorphisms COMT, FUT2, and MTHFR

**DOI:** 10.1016/j.clinsp.2024.100549

**Published:** 2024-12-05

**Authors:** Pedro Andrade, Aline Boveto Santamarina, Jéssica Alves de Freitas, Annete Bressan Rente Ferreira Marum, Ana Flávia Marçal Pessoa

**Affiliations:** a Instituto Medicina e Nutrição de Precisão, São Paulo, Brazil; b Laboratório de Produtos e Derivados Naturais, Laboratório de Investigação Médica-26 (LIM-26), Departamento de Cirurgia, Faculdade de Medicina da Universidade de São Paulo, São Paulo, SP 01246903, Brazil; c Paulista School of Medicine, Federal University of São Paulo - UNIFESP, São Paulo, SP 04021-001, Brazil; d Laboratório de Parasitologia Médica (LIM-46), Departamento de Doenças Infecciosas e Parasitárias, Faculdade de Medicina da Universidade de São Paulo, São Paulo, SP 05403-000, Brazil; e Botânio Pesquisa e Desenvolvimento Ltda, São Paulo, SP 05545010, Brazil

**Keywords:** Single nucleotide polymorphism, FUT2 gene, COMT gene, MTHFR gene, Perimenopause, Vitamins B complex

## Abstract

This mini-review explores the potential of precision medicine and personalized nutrition in addressing health challenges faced by perimenopausal women, focusing on the role of genetic polymorphisms in key metabolic pathways. Specifically focus on the single nucleotide polymorphisms (SNPs) in the COMT, FUT2, and MTHFR genes, which influence neurotransmitter metabolism, gut microbiota composition, and folate homeostasis, respectively. These polymorphisms are critical in modulating hormonal fluctuations, metabolic imbalances, and nutrient absorption during perimenopause. The review highlights the impact of COMT rs4680 on stress response and mood disorders, FUT2 rs602662 and rs601338 on vitamin B12 absorption and cortisol metabolism, and MTHFR rs1801133 and rs1801131 on homocysteine levels and cardiovascular risk. Furthermore, the integration of machine learning in precision medicine is discussed, offering insights into how genetic data can optimize personalized interventions. This approach enables targeted nutritional and therapeutic strategies to mitigate the metabolic and psychological effects of perimenopause. Overall, this review underscores the importance of incorporating genetic testing into preventive care for perimenopausal women to enhance quality of life and promote healthy aging.

## Introduction

Precision, individualized, and preventive medicine has gained increasing recognition for identifying genetic predispositions that influence health throughout different stages of life. Understanding the underlying mechanisms is crucial for developing personalized strategies aiming to correct nutritional deficiencies and optimize physiological responses in specific populations.^1^

Single nucleotide polymorphisms (SNPs) are variations in a single nitrogenous base within the DNA sequence at specific genomic positions. These polymorphisms are the most common form of genetic variation among individuals and can affect susceptibility to diseases, drug responses, and other phenotypic traits.^2^ While many SNPs are neutral, others have significant functional implications, altering gene expression or protein structure and function. As such, SNPs are of substantial interest in genetic research and precision medicine, where their role in health and the potential for personalized therapies is a focus for disease prevention.^3^^,^^4^

Preventive medicine seeks to identify and mitigate disease risks before clinical manifestations arise. In the context of genetic polymorphisms, understanding these variants enables early interventions, such as tailored clinical treatments or lifestyle modifications, which can help to prevent or reduce future health complications.^1^^,^^5^ The cost-effectiveness of genetic testing becomes particularly acceptable from a long-term perspective, as preventing chronic diseases can significantly reduce healthcare costs while improving life expectancy and quality of life.^6^

During perimenopause—the transitional phase preceding menopause—women experience a range of symptoms due to hormonal fluctuations, particularly in estrogen and progesterone levels.^7^ Common symptoms include hot flashes, night sweats, menstrual irregularities, libido changes, mood swings, and insomnia, all of which can severely impact the quality of life, both physically and emotionally.^7^ The COMT, FUT2, and MTHFR genes are key players in the physiology of perimenopausal women, and genetic variants in these genes can profoundly affect their health and quality of life.^8^, ^9^, ^10^ Understanding these genetic and metabolic interactions is essential for the design of personalized nutritional and therapeutic interventions that can alleviate the challenges of this transition, supporting improved quality of life and healthy aging.

The FUT2 gene (fucosyltransferase 2) encodes an enzyme crucial for the synthesis of fucooligosaccharide antigens, which are expressed in various human secretions, including saliva, mucus, and gastrointestinal fluids. Polymorphisms at the rs602662 and rs601338 loci of the FUT2 gene are of significant interest in clinical research, as they directly affect the enzyme's activity, influencing an individual's secretor or non-secretor status.^11^ In perimenopausal women, hormonal fluctuations can impact nutrient metabolism and endocrine balance, increasing susceptibility to metabolic imbalances.^8^ Polymorphisms in FUT2, such as those associated with rs602662 and rs601338, may lead to reduced or altered production of intrinsic factors, compromising vitamin B12 absorption and resulting in deficiency. FUT2 plays a key role in synthesizing intrinsic factor, a glycoprotein produced by stomach parietal cells that binds to vitamin B12, facilitating its absorption in the ileum.^11^ Women with reduced FUT2 activity due to these polymorphisms may experience altered gut microbiota, impairing vitamin B12 absorption and leading to deficiencies that affect energy metabolism, DNA synthesis, red blood cell formation, and neurological function.^12^

In addition, FUT2 polymorphisms may indirectly influence cortisol metabolism. Cortisol, a hormone essential for stress response and the regulation of carbohydrate, protein, and lipid metabolism, may exhibit increased volatility during perimenopause.^7^^,^^13^ The gut microbiota, modulated by FUT2, affects cortisol production and regulation through the hypothalamic-pituitary-adrenal (HPA) axis. Polymorphisms in FUT2 may impair stress response efficiency, exacerbating perimenopausal symptoms such as anxiety, sleep disturbances, and mood swings.^13^

The MTHFR gene (methylenetetrahydrofolate reductase) encodes an enzyme critical for folate metabolism, which plays a central role in the methylation cycle and in maintaining methyl group homeostasis. Methylenetetrahydrofolate reductase converts methylenetetrahydrofolate to methyltetrahydrofolate, the active form of folate necessary for remethylating homocysteine into methionine, a precursor for synthesizing essential biomolecules, including proteins and neurotransmitters. This process is vital for DNA synthesis, neurotransmitter production, and regulation of homocysteine levels, an amino acid whose elevation is associated with increased risks for cardiovascular disease and metabolic disorders.^14^

The most studied polymorphisms in the MTHFR gene are rs1801131 and rs1801133, which can result in reduced MTHFR activity, affecting folate metabolism and the subsequent metabolism of other vitamins and biomolecules.^10^ Decreased MTHFR activity impairs homocysteine-to-methionine conversion, leading to homocysteine accumulation in plasma. Elevated homocysteine levels are associated with heightened risks for cardiovascular disease and neurological complications.^15^ Folate metabolism and homocysteine regulation also have implications for HPA axis function, which governs cortisol production. Folate deficiencies and elevated homocysteine levels can negatively impact the stress response and cortisol regulation, as proper methylation is critical for neuroendocrine and hormonal function.^16^

In perimenopausal women, the hormonal fluctuations of this life stage may interact with MTHFR polymorphisms, influencing cortisol production and stress response. This interaction could exacerbate symptoms like mood swings and fatigue, which are common during perimenopause.^10^^,^^17^ Similarly, the COMT gene (catechol-O-methyltransferase) is vital for the metabolism of catecholamines, including neurotransmitters such as dopamine, epinephrine, norepinephrine, and estrogens. These hormones are central to mood regulation, stress response, and hormonal balance. COMT catalyzes the methylation of catecholamine hydroxyl groups, a key step in their inactivation and clearance from the body. Polymorphisms in the COMT gene, such as rs4680 and rs4633, alter enzyme activity, thereby influencing the concentration and effectiveness of neurotransmitters and hormones in circulation.^18^

The rs4680 polymorphism results in the substitution of valine with methionine at position 158 of the COMT protein, leading to a reduction in catechol-O-methyltransferase enzymatic activity.^19^ This reduction can result in elevated levels of dopamine in the brain, which, while potentially beneficial for cognitive function in certain contexts, may also predispose individuals to heightened stress reactivity and mood disorders.^20^ In perimenopausal women, where hormonal fluctuations are pronounced, changes in COMT activity can intensify symptoms such as anxiety, depression, and mood swings—conditions often linked to variations in catecholamine and estrogen levels.^9^ Additionally, COMT indirectly influences nutrient metabolism, particularly the metabolism of vitamin D and stress hormones such as cortisol.^18^

Perimenopausal women carrying this polymorphism may experience a dampened stress response due to catecholamine accumulation, potentially disrupting cortisol regulation. Cortisol, in turn, plays a critical role in the metabolism of various vitamins, including vitamin D, which is vital for bone health and immune balance. Fluctuations in cortisol levels, modulated by COMT activity, may compromise vitamin D homeostasis, increasing the risk of deficiency—a growing concern for perimenopausal women.^21^^,^^22^ During perimenopause, hormonal balance is particularly sensitive to changes in neurotransmitter metabolism.^23^ Reduced COMT activity can exacerbate stress-related symptoms and hormonal imbalances, contributing to mood disorders and cortisol dysregulation.^21^ Inadequate cortisol regulation can manifest as fatigue, mood instability, and challenges with weight management.^9^

The imbalance in catecholamine metabolism caused by COMT polymorphisms can also disrupt the utilization of metabolic cofactors, such as folate and vitamin B12, intertwining with the metabolic pathways of the FUT2 and MTHFR genes. Although the direct impact on vitamin concentrations may not be immediately evident, the broader effect on overall metabolism can have significant implications for health and nutritional balance. Genetic variants in COMT, FUT2, and MTHFR may exert substantial effects on the health and quality of life of perimenopausal women.^8^, ^9^, ^10^ These variants can alter hormonal regulation, nutrient absorption, and neurotransmitter metabolism, contributing to the severity and expression of symptoms during this transitional life stage.^7^^,^^24^ Understanding the genetic influences on these processes is essential for the development of personalized precision medicine approaches to symptom management and health promotion during perimenopause.

Given the complexity of this research field, machine learning offers substantial advantages by enabling the analysis of large volumes of genetic data that would be difficult to interpret using traditional statistical methods.^25^ Machine learning allows for the investigation of interactions between single nucleotide polymorphisms (SNPs) and their associations with clinical traits or other variables of interest, such as disease risk. This approach can uncover subtle and intricate patterns that conventional statistical techniques may fail to detect.^26^ As a result, machine learning facilitates the development of predictive models that can assess genetic predispositions, identify potential therapeutic targets, and personalize treatments based on an individual's genetic profile. In addition to its clinical applications, machine learning accelerates discovery in genomic research, enabling the identification of novel associations between SNPs and phenotypes, and ultimately advancing the field of precision medicine.^3^^,^^26^ These techniques enhance the interpretation of genetic data and enable the integration of complex information into personalized care strategies, optimizing healthcare practices to be more efficient and tailored to individual needs.^4^^,^^27^

By exploring the interactions between genetics and nutritional biomarkers, this study aims to provide valuable insights into the mechanisms driving metabolic and nutritional changes during perimenopause. Such findings could inform the development of personalized preventive strategies, improving the health and well-being of women during this critical life stage.

## Relevant genetic polymorphisms in perimenopause

The COMT (catechol-O-methyltransferase) gene encodes a crucial enzyme involved in the metabolism of catecholamines, including dopamine, norepinephrine, and epinephrine. This enzyme plays a vital role in the degradation of these neurotransmitters, regulating their synaptic concentrations and modulating physiological processes such as mood, stress response, and cognitive function.^28^ The rs4680 polymorphism, also known as Val158Met, results in a substitution of valine (Val) with methionine (Met) at position 158 of the protein, leading to a reduction in COMT activity by approximately 25% to 40%.^20^ Women carrying the Met allele tend to exhibit increased sensitivity to catecholamines, which may influence mood regulation and elevate the risk of developing anxiety or depression, particularly during perimenopause, when significant hormonal fluctuations occur.^22^

At the cellular level, the COMT enzyme operates in the prefrontal cortex, a brain region critical for executive functions and emotional regulation. The reduced COMT activity associated with the Met allele may result in prolonged dopamine persistence within this region, enhancing synaptic plasticity.^29^ However, this may also increase vulnerability to oxidative stress due to the accumulation of catecholamines and their metabolites. Oxidative stress is a key contributor to chronic inflammation and degenerative processes, which can become more pronounced during the menopausal transition as estrogen levels decline—a hormone known for its antioxidant properties. Furthermore, the interaction between the rs4680 polymorphism and fluctuating hormone levels in perimenopause may affect energy metabolism and thermoregulation, contributing to vasomotor symptoms such as hot flashes.^30^ In this context, targeting the modulation of COMT activity could be a promising approach for personalized interventions aimed at mitigating the psychological and physical challenges women face during this transitional phase of life.

The FUT2 gene (fucosyltransferase 2) is involved in the production of blood group antigens within the intestinal epithelium and plays a fundamental role in shaping the gut microbiota.^11^ The rs601338 polymorphism in the FUT2 gene is linked to non-secretor status, wherein individuals homozygous for the mutant allele (AA) do not produce antigens in the intestinal mucus, leading to alterations in microbiota composition.^12^ In perimenopausal women, the influence of this polymorphism may be particularly significant, as the hormonal changes associated with menopause also impact the gut microbiota.^31^ Research suggests that non-secretors have a more diverse microbiota that may be more resistant to dysbiosis.^32^ However, this variation is also linked to an increased risk of vitamin deficiencies, particularly vitamin B12, as gut bacteria rely on secreted antigens for proper colonization of the intestines.^33^ A deficiency in vitamin B12, common among non-secretors, may exacerbate symptoms such as fatigue and cognitive impairment during perimenopause.

Mechanistically, alterations in antigen production and the subsequent modulation of the microbiota directly influence the intestinal barrier and the communication between the immune system and the microbiome. The gut microbiota plays a pivotal role in regulating pro-inflammatory cytokines, which are often elevated in perimenopausal women, contributing to systemic inflammation and metabolic disturbances, such as weight gain and insulin resistance.^34^ The FUT2 rs601338 polymorphism may thus have important implications for immunometabolism regulation, suggesting that women with this genotype could benefit from personalized probiotic or prebiotic interventions to mitigate the adverse effects of this hormonal transition.

The MTHFR (methylenetetrahydrofolate reductase) gene encodes an enzyme essential for folate metabolism, specifically in the conversion of homocysteine to methionine—a critical step in the methylation cycle.^35^ Two well-studied polymorphisms, rs1801131 (A1298C) and rs1801133 (C677T), are both associated with reduced MTHFR enzyme activity. For instance, the T allele at rs1801133 can reduce enzyme activity by up to 70% in homozygous individuals, resulting in elevated plasma homocysteine levels, a known marker of inflammation and cardiovascular risk.^36^

During perimenopause, the risk of cardiovascular disease is already heightened due to declining estrogen levels.^37^ Elevated homocysteine levels, resulting from reduced MTHFR activity, may further exacerbate this risk. Moreover, hyperhomocysteinemia is associated with an increased likelihood of cognitive disorders, including dementia and Alzheimer's disease, making polymorphisms in this gene particularly relevant to brain health during aging.^38^ At the cellular level, excessive homocysteine induces oxidative stress, contributing to endothelial dysfunction and compromising the integrity of blood vessels.^37^ A reduced methylation capacity also impairs neurotransmitter synthesis, influencing both mood and cognitive function.^39^ Interventions aimed at improving folate status and lowering homocysteine levels are particularly important for women with these polymorphisms, as they may help mitigate the effects of hormonal decline and protect against adverse cardiovascular and mental health outcomes. The identification and understanding of polymorphisms in COMT, FUT2, and MTHFR provide valuable insights for the personalization of nutritional and therapeutic strategies in perimenopausal women. The interaction of these genes with the hormonal and metabolic environment during this phase of life highlights the importance of precision medicine, tailoring interventions to individual genetics to optimize health and well-being as women age.

## Polymorphisms on nutrient metabolism

Though small, these genetic changes of SNPs can significantly influence the bioavailability of vitamins, minerals, and other nutrients by modulating their absorption, transport, storage, and cellular utilization.^40^ In the context of perimenopause, a phase marked by hormonal fluctuations that affect metabolism, understanding the role of SNPs is particularly important, as maintaining nutritional homeostasis is critical to mitigating the adverse effects commonly associated with this stage.

The MTHFR gene polymorphisms such as rs1801133 (C677T) and rs1801131 (A1298C) are well-studied and have been linked to elevated plasma homocysteine levels and decreased capacity for folate regeneration in its active form.^10^ In perimenopausal women, this disruption may heighten cardiovascular disease risk, as estrogen's protective effects on the cardiovascular system begin to wane.^31^ Furthermore, folate deficiency, stemming from reduced MTHFR activity, may impair the metabolism of other B vitamins, such as B6 and B12, exacerbating the common nutritional deficiencies seen during this stage.^41^

Polymorphisms such as rs601338 and rs602662 in FUT2 gene affect the expression of the "secretor" or "non-secretor" phenotype, which has profound implications for the gut microbiota and, subsequently, nutrient metabolism. Non-secretors, for instance, have lower diversity in their gut bacteria, especially those that use ABO antigens as substrates, such as certain *Bifidobacterium* strains.^12^ These bacteria are essential for fermenting dietary fiber and producing short-chain fatty acids, crucial for maintaining intestinal barrier integrity and immune regulation.^42^ The altered microbiota profile in non-secretors can compromise the absorption of micronutrients like vitamin D and calcium, which are vital for bone health and inflammation control—particularly important for women transitioning into menopause.^17^

The COMT gene, encoding catechol-O-methyltransferase, is involved in the degradation of catecholamines and estrogens and also impacts nutrient metabolism, especially during perimenopause, when estrogen fluctuations increase the risk of metabolic and cognitive dysfunctions.^9^ Reduced COMT activity extends the half-life of catecholamines and estrogens, potentially offering a protective effect against cognitive decline by maintaining higher estrogen levels in the brain.^19^ However, it also raises the risk of accumulating genotoxic estrogen metabolites, such as catechol estrogens, which can damage DNA and elevate breast cancer risk.^31^ Furthermore, diminished COMT activity is associated with impaired stress response regulation, indirectly affecting nutrient metabolism. Chronic catecholamine elevation, particularly of norepinephrine and dopamine, can exacerbate systemic inflammation, promoting insulin resistance, and impair glucose utilization.^22^ It may also hinder the absorption of essential minerals like magnesium and zinc, which are critical cofactors in energy metabolism and immune function. Magnesium, in particular, is vital for over 300 biochemical reactions in the body, and its deficiency has been linked to mitochondrial dysfunction and chronic fatigue.^43^

These SNPs clearly do not act in isolation; they interact within complex networks involving metabolic pathways, the gut microbiome, and hormonal and thyroid homeostasis.^3^ Polymorphisms in genes like MTHFR, FUT2, and COMT significantly alter nutrient metabolism and utilization, predisposing individuals to specific health conditions such as cardiovascular disease, cognitive dysfunction, and metabolic disorders.^8^, ^9^, ^10^ In perimenopausal women, these effects are further intensified by the natural decline in estrogen, which itself impacts nutrient balance and energy metabolism.^8^

Thus, identifying these polymorphisms within the context of precision medicine allows for the personalization of nutritional interventions, aimed at compensating for metabolic deficiencies and mitigating the risks associated with aging and the hormonal changes of perimenopause. Personalized strategies can optimize health and well-being during this critical stage of life.

## Personalized nutritional approaches

Personalized nutrition represents a key aspect of precision medicine, incorporating genetic, metabolomic, and microbiome data to optimize nutritional status and prevent disease.^4^ In the context of genetic polymorphisms such as MTHFR, FUT2, and COMT, tailored nutritional interventions become essential tools to modulate physiological and metabolic responses in at-risk populations, particularly perimenopausal women—a phase marked by significant hormonal, inflammatory, and metabolic changes.

The MTHFR gene plays a crucial role in the folate cycle and methylation processes. Since folic acid, the inactive form of folate, requires MTHFR-mediated conversion to its active form, methylfolate, individuals with these polymorphisms may exhibit inadequate folate availability for DNA synthesis, cellular repair, and neurotransmitter methylation.^14^ In such cases, personalized supplementation with 5-methyltetrahydrofolate (5-MTHF)—the biologically active form—can bypass the enzymatic limitation, ensuring sufficient tissue folate and lowering homocysteine levels.^16^ This approach is particularly relevant for perimenopausal women, who face increased cardiovascular risk due to both elevated homocysteine and hormonal decline.^14^

Impaired folate metabolism also disrupts the methionine cycle, reducing the production of S-adenosylmethionine (SAMe), a critical methyl donor involved in hundreds of biochemical reactions. Low SAMe levels can affect mental health and lipid metabolism, as methylation is crucial for neurotransmitter regulation and phospholipid synthesis.^36^ Therefore, personalized nutritional strategies for MTHFR polymorphism carriers may also include increased intake of B vitamins (B6 and B12), which synergistically support the conversion of homocysteine to methionine, optimizing the methylation cycle.^17^

In individuals with FUT2 gene polymorphisms, such as rs601338 and rs602662, modulating the gut microbiota becomes critical. The absence of fucosylation in non-secretors affects the composition and diversity of the microbiota, hindering the colonization of beneficial bacterial strains producing short-chain fatty acids, such as butyrate, which are essential for maintaining intestinal barrier integrity and modulating immune responses.^12^ Personalized nutritional strategies for non-secretors should include prebiotics and probiotics targeted to support a healthy microbiota. Supplementation with specific *Bifidobacterium* and *Lactobacillus* strains can enhance gut health and improve micronutrient absorption, particularly vitamin D, which tends to be deficient in non-secretors.^32^ Prebiotic fibers like galactooligosaccharides (GOS) and fructooligosaccharides (FOS) can further promote beneficial bacterial growth, bolstering intestinal defense and optimizing mineral absorption.^44^, ^45^

Another important consideration for non-secretors is vitamin B12 absorption. Research shows that FUT2 polymorphism carriers are more likely to develop B12 deficiency, given the role of the microbiome in B12 metabolism.^33^ Supplementation with methylcobalamin, the active form of B12, may effectively counteract this deficiency, particularly in perimenopausal women, who may experience increased B12 requirements due to cognitive and metabolic changes.^10^

For individuals with the COMT gene rs4680 polymorphism (Val158Met), a personalized nutritional approach should prioritize estrogen metabolism and oxidative stress regulation.^22^ Reduced COMT activity in Met variant carriers leads to the accumulation of catechol estrogens, raising the risk of DNA damage from oxidative stress.^29^ Nutritional interventions could include specific antioxidants like polyphenols, which neutralize reactive oxygen species produced during estrogen metabolism.^46^ Quercetin, found in onions and apples, can inhibit the formation of harmful DNA adducts by catechol estrogens, providing protection against oxidative damage.^47^ Additionally, silymarin, a flavonoid from milk thistle, has demonstrated hepatoprotective effects and may help mitigate oxidative stress in the liver, the main organ responsible for steroid hormone metabolism.^48^

In terms of neurotransmitter metabolism, L-tyrosine supplementation may benefit individuals with the COMT Met variant. As a dopamine precursor, L-tyrosine can help compensate for reduced COMT activity, improving mood and stress regulation^28^—common challenges during perimenopause. Furthermore, vitamin D supplementation is particularly important for women with COMT polymorphisms, as it influences not only immune function and bone health but also neurotransmitter synthesis.^20^ Optimized vitamin D intake may provide neuroprotection and mitigate hormonal fluctuations experienced during perimenopause.

Thus, personalized nutritional interventions based on genetic polymorphisms offer a promising approach to enhancing health and reducing disease risk associated with aging.^5^ The targeted use of nutraceuticals and supplements tailored to individual genetic profiles allows for more precise modulation of nutrient metabolism, inflammation, and oxidative stress, ultimately improving the quality of life for women during perimenopause.

## Machine learning and precision medicine

The integration of machine learning (ML) into precision medicine has transformed the analysis of large, complex datasets, enhanced the accuracy of predictions and enabled more personalized interventions based on patients’ genetic, metabolic, and phenotypic profiles.^27^ In the context of genetics and nutrition, ML applications uncover patterns and associations between genetic polymorphisms, environmental factors, and lifestyle variables, making it a critical tool for improving prevention and treatment strategies in perimenopausal women.

The foundation of ML in precision medicine lies in the analysis of genomic data and its interaction with the environment.^26^ Single nucleotide polymorphisms (SNPs) in genes like MTHFR, COMT, and FUT2 significantly influence nutrient metabolism, hormonal regulation, and inflammatory responses.^8^, ^9^, ^10^ ML algorithms allow for the prediction of how variations in these genes impact susceptibility to conditions such as cardiovascular disease, metabolic disorders, and mood disturbances during perimenopause.

Supervised learning models, such as Random Forests, Support Vector Machines (SVM), and Artificial Neural Networks, are frequently used to correlate polymorphisms with clinical outcomes by learning from labeled datasets.^25^ For example, variants in the MTHFR gene, which affect folate metabolism, can be analyzed alongside data on homocysteine levels, inflammation, and energy metabolism.^14^ These models help predict which individuals are at higher risk for hyperhomocysteinemia, a condition linked to increased cardiovascular risk, and identify the most effective nutritional interventions. Tailored strategies can adjust doses of 5-methyltetrahydrofolate or essential cofactors like vitamins B6 and B12 based on individual enzyme activity.

ML also excels in analyzing metabolomic data and gut microbiota composition. Through unsupervised clustering techniques, ML can classify patients by microbial profiles, identifying those prone to dysbiosis, systemic inflammation, and impaired nutrient absorption.^49^ Predictive models guide personalized interventions, such as targeted prebiotic and probiotic therapies, to enhance the gut environment and improve the metabolism of micronutrients like vitamins D and B12, which are commonly deficient in FUT2 non-secretor individuals.

Artificial neural networks have proven particularly valuable for modeling complex gene-environment interactions.^25^ Women with the Met variant in COMT gene may exhibit higher catecholamine accumulation, increasing their risk for mood disorders and oxidative stress.^20^ ML algorithms can integrate data on hormone levels, oxidative stress biomarkers, and genetic variants to predict individual outcomes. Such insights enable interventions targeting neurotransmitter modulation, such as L-tyrosine supplementation, and the use of antioxidants like polyphenols to mitigate oxidative stress.

Moreover, ML techniques like time series analysis and linear regression models provide a deeper understanding of how temporal changes in micronutrient levels, hormones, and inflammatory markers correlate with genetic variations.^4^^,^^27^ Monitoring homocysteine levels in women with MTHFR polymorphisms may indicate the need for increased methylfolate supplementation during periods of heightened metabolic demand, such as the perimenopausal transition, thus preventing cardiovascular complications.

Reinforcement learning represents a novel approach in optimizing nutritional interventions.^27^ These algorithms learn from feedback, dynamically adjusting supplement recommendations based on individual physiological responses.^49^ This capability is particularly valuable in fine-tuning doses of folate, vitamin B12, and vitamin D over time, adapting to the hormonal and metabolic fluctuations characteristic of perimenopause. The continuous refinement of recommendations based on real-time responses is a key advantage of ML in precision medicine, enabling personalized care to evolve in response to changing patient needs.^50^

ML's role extends beyond personalized interventions, offering significant potential for risk stratification and treatment response prediction.^6^ Individuals with MTHFR, FUT2, and COMT variants can be categorized into high- or low-risk groups for conditions like cardiovascular disease, cognitive dysfunction, or nutrient deficiencies. This stratification enhances the precision of interventions, reducing the reliance on generalized treatments that may be ineffective or even detrimental in certain populations.^5^ Additionally, ML-based risk stratification improves the cost-effectiveness of healthcare by concentrating efforts where they are most needed.^6^

The use of machine learning in precision medicine offers a sophisticated approach to unraveling the complexities of genetic, metabolic, and environmental interactions.^49^ By integrating genetic data with phenotypic and lifestyle information, ML algorithms enable more precise personalization of nutritional and therapeutic interventions, especially for at-risk populations like perimenopausal women. This technology has the potential to optimize disease prevention and treatment, advancing a truly individualized approach to healthcare.^4^

## Future perspectives and conclusion

**Fig. 1:**
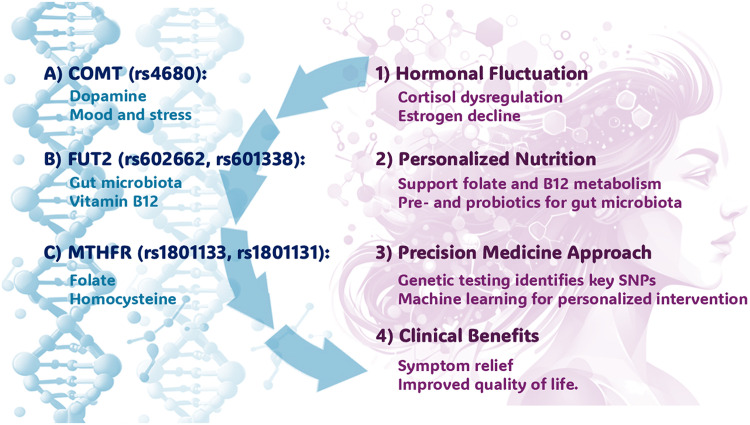
The impact of genetic polymorphisms in the COMT (rs4680), FUT2 (rs 602,662, rs601338), and MTHFR (rs1801133, rs1801131) genes on nutrient metabolism and hormonal regulation is particularly significant during perimenopause. Variations in these genes influence mood, stress responses, gut microbiota, and the absorption of essential nutrients like folate and vitamin B12. To address these effects, personalized nutritional interventions are recommended. The integration of precision medicine, utilizing genetic testing and machine learning, aims to enhance symptoms and improve the quality of life for affected women.

The advancement of precision medicine, particularly regarding the personalized nutrition and genomics, heralds a promising future for technological and scientific innovations.^1^ Ongoing progress in the “omics” analysis like genomic, metabolomic, and microbiome data, combined with the machine learning and artificial intelligence application, paves the way for truly individualized medical care.^49^ By delving deeper into the interactions among genetic polymorphisms, environmental factors, and epigenetic influences, we can develop highly targeted preventive and therapeutic interventions for specific populations, such as perimenopausal women, who experience unique physiological and metabolic changes (Fig. 1).

From a cellular and molecular perspective, the complexity of gene-environment interactions continues to evolve. The integration of cutting-edge technologies, such as CRISPR gene editing and next-generation sequencing (NGS), will enhance our ability to elucidate the mechanisms through which single nucleotide polymorphisms (SNPs) impact critical cellular pathways.^2^ For instance, variants in the MTHFR, COMT, and FUT2 genes represent only a portion of the factors influencing metabolic and inflammatory regulation in perimenopausal women. Understanding how these SNPs interact with other genes via intricate regulatory networks is crucial for optimizing interventions.^3^ While the effects of SNPs on folate metabolism,^10^^,^^14^^,^^15^^,^^41^ neurotransmitter degradation,^13^^,^^18^^,^^19^ and gut microbiota^12^^,^^31^^,^^51^ are well-documented, exploring their interplay with epigenetic factors, such as DNA methylation and histone modification, holds promise. Future research should aim to characterize these interactions and their implications for chronic diseases like cardiovascular disease, obesity, type 2 diabetes, and mood disorders, which are prevalent in perimenopausal women. Advanced ML models, including deep learning and convolutional neural networks, will be essential for uncovering nonlinear patterns that traditional statistical methods may miss, such as non-additive gene interactions where one polymorphism influences the expression or function of another gene.^25^

The concept of modulating the gut microbiota through personalized nutritional interventions will become increasingly integrated into precision healthcare.^40^ Predicting which bacterial strains will respond optimally to prebiotics or probiotics based on an individual's genetic and microbiomics profile offers a promising avenue for future therapeutic strategies.^49^ Additionally, advances in synthetic biology and genome therapy offer potential for correcting harmful polymorphisms, such as those in the MTHFR gene, which affect methylation and folate metabolism. While this approach could address diseases like cardiovascular and cognitive disorders,^51^ caution is necessary due to potential gene interactions, pleiotropic effects, and complex gene interactions arising from genome modifications.^4^

From a clinical perspective, personalized interventions will not only enhance treatment efficacy but also improve patient adherence, as treatments will be tailored to individual needs. Real-time adjustments to precision nutrition guidelines based on continuous metabolic, inflammatory, and hormonal biomarker data will allow for more dynamic and flexible interventions, adapting swiftly to the physiological changes associated with perimenopause and mitigating the risk of adverse outcomes.^1^^,^^3^

Looking ahead, a significant challenge will be integrating these personalized approaches into public health systems, particularly in resource-limited settings. Ensuring equitable access to genetic sequencing technologies and ML tools is crucial to distributing the benefits of precision medicine.^5^^,^^6^ Public health programs focused on disease prevention for at-risk groups, such as perimenopausal women, could greatly benefit from personalized interventions based on genomic and metabolomic data.

Thus, the advancement of precision medicine, driven by machine learning and the integration of omics data, promises to revolutionize our understanding and treatment of human health. Personalizing interventions based on individual genetic and metabolic profiles will enable a more effective and targeted approach to preventing and treating chronic diseases. Despite existing technical and ethical challenges, the future of precision medicine appears promising, offering the potential for truly individualized healthcare tailored to the unique needs of each patient, particularly in vulnerable populations like perimenopausal women.

## Conflict of Interests

The authors declare no competing interests.
